# Analysis of Cause of Endodontic Failure of C-Shaped Root Canals

**DOI:** 10.1155/2018/2516832

**Published:** 2018-11-25

**Authors:** Yemi Kim, Donghee Lee, Da-Vin Kim, Sin-Young Kim

**Affiliations:** ^1^Department of Conservative Dentistry, Dental Research Institute, Ewha Womans University School of Medicine, Seoul, Republic of Korea; ^2^Department of Conservative Dentistry, Seoul St. Mary's Dental Hospital, College of Medicine, The Catholic University of Korea, Seoul, Republic of Korea

## Abstract

The purpose of this study was to analyze various characteristics and classification of C-shaped root canals and evaluate the causes of endodontic failure of C-shaped root canals by examining the resected root surface with an endodontic microscope and a scanning electron microscope (SEM). Forty-two teeth with C-shaped root canals were included in this study and had undergone intentional replantation surgery. Before surgery, periapical radiography and cone-beam computed tomography were taken. The root canal configuration was analyzed and classified according to Melton's classification at coronal and apical level. After injection of 1 : 100,000 epinephrine with 2% lidocaine, the tooth was carefully extracted. After the root-end resection, the resected root surface was examined using an operating microscope and SEM. Mandibular second molars were most frequently involved teeth (90.4%). The most frequently observed root canal configurations were C1 at the coronal level (45.2%) and C3 at the apical 3 mm level (45.2%). The most common cause of failure for a C-shaped root canal treatment was a leaky canal (45.2%), followed by an isthmus (23.8%), missing canal, overfilling, and iatrogenic problems. In conclusion, C-shaped root canals were most frequently found in mandibular second molars. The most common cause of failure was a leaky canal and isthmus.

## 1. Introduction

The C-shaped root canal system is an anatomical variant of the root canal structure, whose characteristic feature is the connection of the root canals by a fin or web-like structure to form a C shape at the root canal orifice [1]. The term C-shaped canal was first introduced by Cooke and Cox [2] in 1979 to describe the cross-sectional morphology of roots that resembled letter C. A high prevalence of C-shaped root canals has been reported in mandibular second molars in the Asian population [3–6]. However, they can also be found in maxillary first molars [7–9], maxillary second molars [10, 11], and mandibular first molars [12, 13].

Causes of endodontic failure can be classified into biological and technical factors. Failures related to microorganisms can be caused by anatomical difficulties such as isthmus, apical ramification, and other morphological irregularities [14]. The complexity of C-shaped canals makes them difficult to clean, shape, and obturate effectively [15, 16]. Failures also can be caused by procedural errors such as root perforation, separated instruments, or missed canals. The thin dentinal wall of the buccal or lingual groove may lead to strip perforation, which poses a considerable threat to tooth prognosis [4].

If orthograde retreatment and endodontic microsurgery of a tooth with apical periodontitis are not feasible, intentional replantation has been considered the last means of retaining a natural tooth that would otherwise be lost to extraction [17–20]. Incorporating contemporary guidelines for tooth replantation and endodontic microsurgery into intentional replantation procedures, recent studies showed that long-term survival rates of intentional replantation were 73–77% [20, 21]. During an intentional replantation procedure, a precise inspection of the resected root surface helps identify the cause of endodontic failure of C-shaped root canal, and scanning electron microscope (SEM) evaluation can show their anatomical effect on the clinical outcome of root canal treatment.

The purpose of this study was to analyze various characteristics and classification of C-shaped root canals and evaluate the causes of endodontic failure of C-shaped root canals by examining the resected root surface with endodontic microscope and SEM.

## 2. Materials and Methods

### 2.1. Case Selection

This study was approved by the Institutional Review Board of Seoul St. Mary's Hospital (KC15RISI0706). From a total of 417 patients who were referred to the Department of Conservative Dentistry for endodontic microsurgery between March 2009 and August 2015, 42 C-shaped root canals that had undergone intentional replantation surgery were included in this study. Teeth were evaluated clinically and radiographically and diagnosed as C-shaped root canals. The detailed inclusion criteria are the following: (i) nonsurgical root canal retreatment or orthograde retreatment failed to relieve pain; (ii) a separated instrument or metal post could not be removed or bypassed; (iii) the calcified canal was not negotiated; (iv) endodontic microsurgery was not feasible because of anatomical structures such as inferior alveolar nerve, maxillary sinus, and dense cortical bone; and (v) previous endodontic microsurgery had failed. If symptomatic apical periodontitis was persisted, then intentional replantation was planned and written informed consent was obtained from the patients. Before surgery, patient age and gender, tooth number, subjective symptoms, mobility, percussion, bite test, and probing depths were recorded. Periapical radiography and cone-beam computed tomography (CBCT) (i-CAT, Imaging Sciences International LLC, Hatfield, PA, USA) were taken with exposure parameters of 120 kV, 47.74 mA, and 20 seconds. All CBCT scans were reformatted with a 0.25 voxel size.

### 2.2. Surgical Procedures

All of the clinical procedures were performed by one endodontic faculty in the Department of Conservative Dentistry, Seoul St. Mary's Dental Hospital. Two weeks before the surgery, the orthodontic separation ring was inserted into the proximal surfaces of the tooth to produce mobility for easy extraction. After the injection of two ampoules of 1 : 100,000 epinephrine with 2% lidocaine, the tooth was carefully extracted. To avoid root fracture and minimize damage to the periodontal ligament, elevators were not used to luxate the tooth. A slow and weak continuous force was applied buccolingually with extraction forceps. After the extraction of the tooth, soft tissue debris on the root surface was cleaned with sterile physiologic saline. The root surface was evaluated using a microscope (Zeiss OPMI Pico; Carl Zeiss, Oberkochen, Germany). If there was a fracture line, the prognosis was explained to the patient and replantation was not performed. If there was no fracture line, routine replantation procedures were performed. The coronal two-thirds of the root surface was covered with saline-soaked wet gauze, and the apical 3 mm of the root tip was sectioned with a diamond point bur (Komet 858; Komet, Rock Hill, SC, USA) under copious irrigation with sterile saline. The root tip was fixed with 4% buffered paraformaldehyde for 24 hours immediately after resection. The resected root surfaces were stained with methylene blue (Canal-seek; eDENT, Seoul, Korea) and inspected with a microscope under 26x magnification to examine the cause of previous endodontic failure and missed anatomic details. The root-end preparation, which extended up to 3 mm into the canal space along the long axis of the root, was made using a diamond point bur. For root-end filling, mineral trioxide aggregate (MTA) (ProRoot MTA, Dentsply, Tulsa, OK, USA) was used. After socket irrigation with sterile saline, the tooth was replanted and a semirigid splint was performed if necessary. The replantation procedure after extraction was performed within 20 minutes to prevent damage of periodontal ligament. A postoperative mouthwash (0.2% chlorhexidine gluconate) and antibiotics were routinely prescribed, and the semirigid splint was removed two weeks later.

### 2.3. Evaluation and Classification of C-Shaped Root Canals

**Figure 1:**
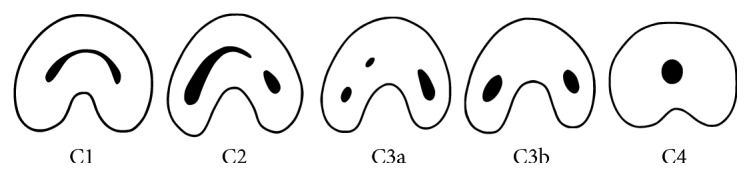
Modified Melton's classification of C-shaped canal configuration [1].

**Figure 2:**
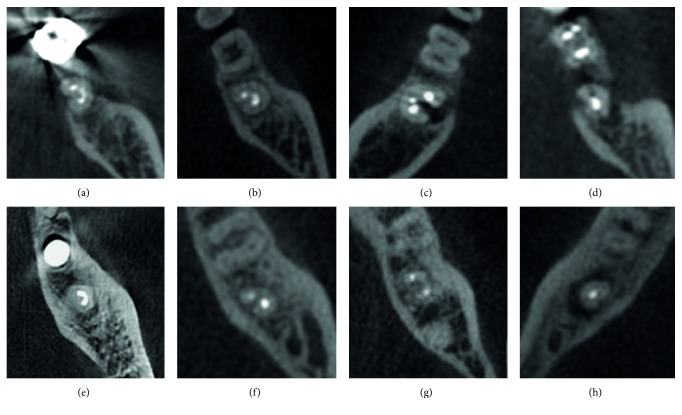
Classification of C-shaped canal configuration using cone-beam computed tomography. (a) C1 configuration at coronal level. (b) C2 configuration at coronal level. (c) C3 configuration at coronal level. (d) C4 configuration at coronal level. (e) C1 configuration at apical 3 mm level. (f) C2 configuration at apical 3 mm level. (g) C3 configuration at apical 3 mm level. (h) C4 configuration at apical 3 mm level.

Three-dimensional images of the tooth that was scheduled for intentional replantation were displayed using Invivo5 dental software (Anatomage, San Jose, CA, USA). The root canal configuration was analyzed and classified according to Melton's classification with the modifications proposed by Fan et al. [1] (Figure 1). The presence of a C-shaped canal system and its configuration was evaluated from the pulp orifice to the apex as axial tomographic slices were viewed at 0.25 mm intervals (Figure 2). The canals were classified as follows:

- C1 (continuous C-shaped canal): C-shaped outline with no separation
- C2 (semicolon-shaped canal): canal configuration in which the dentin separates one distinct canal from another C-shaped buccal or lingual canal
- C3 (separated canals): two or more discrete and separate canals
- C4: a single round or oval canal

During intentional replantation surgery, photos were taken of the root surface, resected root surface, root-end preparation area, and root-end retrograde filling area.

### 2.4. Assessment of Possible Causes of Failure in the Previous Endodontic Treatment

After the root-end resection, the resected root surface was stained with methylene blue and examined carefully to determine the state of the previous endodontic treatment using an operating microscope at 26x magnification. The clinical causes of failure were categorized as follows: (1) leaky canal: a gap between the previous root filling and the dentin or obvious leakage after methylene blue staining; (2) anatomical complexity: isthmus between the two canals filled or an apical ramification that had not been treated; (3) missing canal: untreated canal regardless of the presence of an isthmus; (4) underfilling: fillings more than 2 mm short of the apex in preoperative radiographs; (5) overfilling: excess root filling; (6) iatrogenic problem: perforation, transportation, or file separation; (7) calcified canal; and (8) calculus. For SEM preparation, the resected root tips were immersed in a fixative solution containing 4% buffered paraformaldehyde for 24 hours. The root tips were rinsed with distilled water, dehydrated, then mounted on aluminum stubs, sputter coated with a 30 nm layer of gold, and examined under an S-4700 FESEM (Hitachi, Tokyo, Japan). The voltage was set to 15.0 kV, the signal type was secondary electrons, the working distance was 12 mm, and the scan speed was 16 frames per 20 seconds.

### 2.5. Statistical Analysis

All statistical analyses were performed by Fisher's exact tests using SAS software version 9.2 (SAS Institute Inc., Cary, NC, USA). The level of significance was set at *p* = 0.05.

## 3. Results

**Figure 3:**
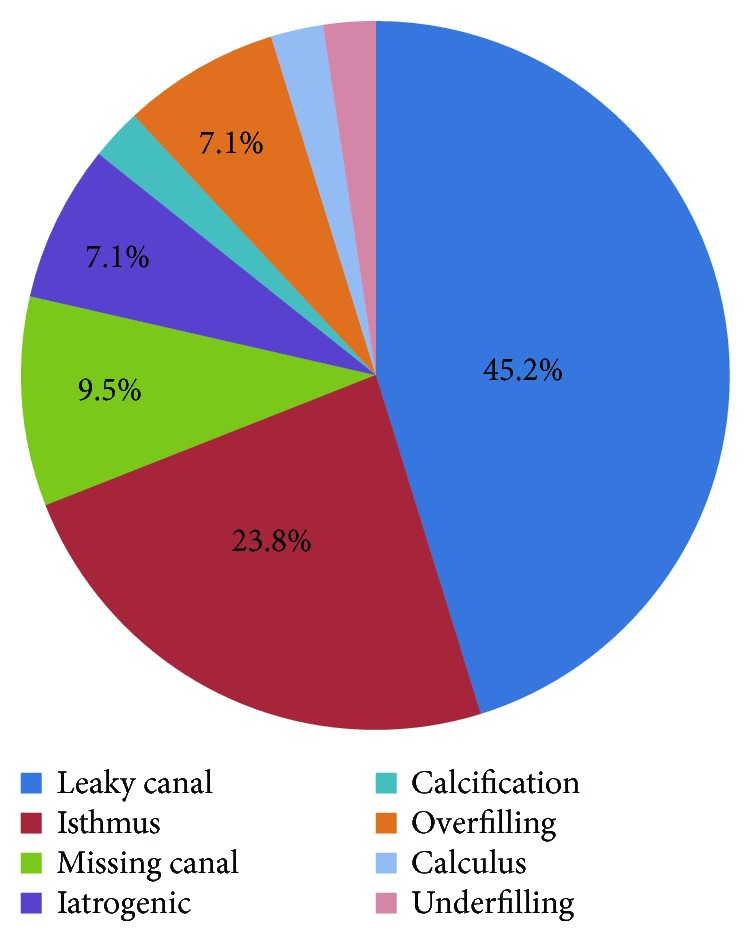
Percentage of the possible causes of failure in the previous endodontic treatment of C-shaped root canals (*p* < 0.05).

**Figure 4:**
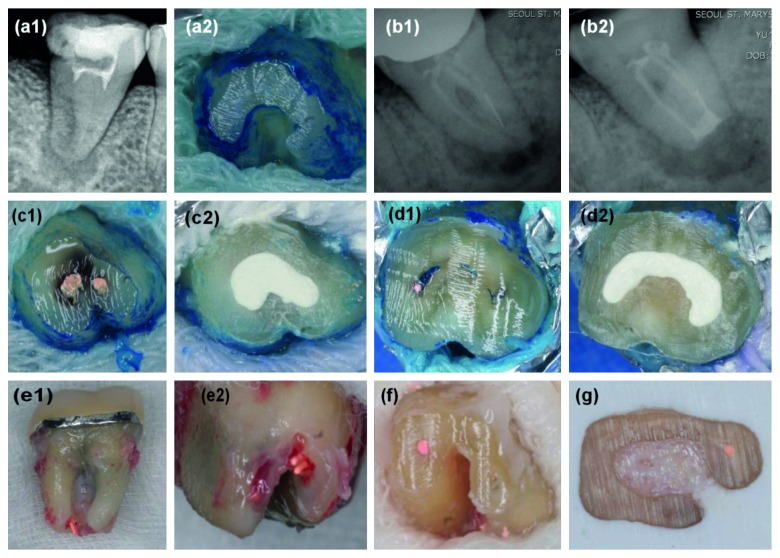
Representative photos of various causes of endodontic failure of C-shaped root canals. (a1) Calcified canal: preoperative periapical radiography. (a2) Root-resected surface of calcified C-shaped root canals. (b1) File separation: preoperative periapical radiography. (b2) Postoperative periapical radiography. (c1) Leaky canal. (c2) Retrograde filling with MTA. (d1) Isthmus connecting mesial and distal canals. (d2) Retrograde filling with MTA. (e1, e2) Overfilling: overextended gutta-percha. (f) Missing canal: only the mesiolingual canal is filled with gutta-percha in the mandibular left second molar. (g) Missing canal: only the palatal canal is filled with gutta-percha in the maxillary right first molar.

**Figure 5:**
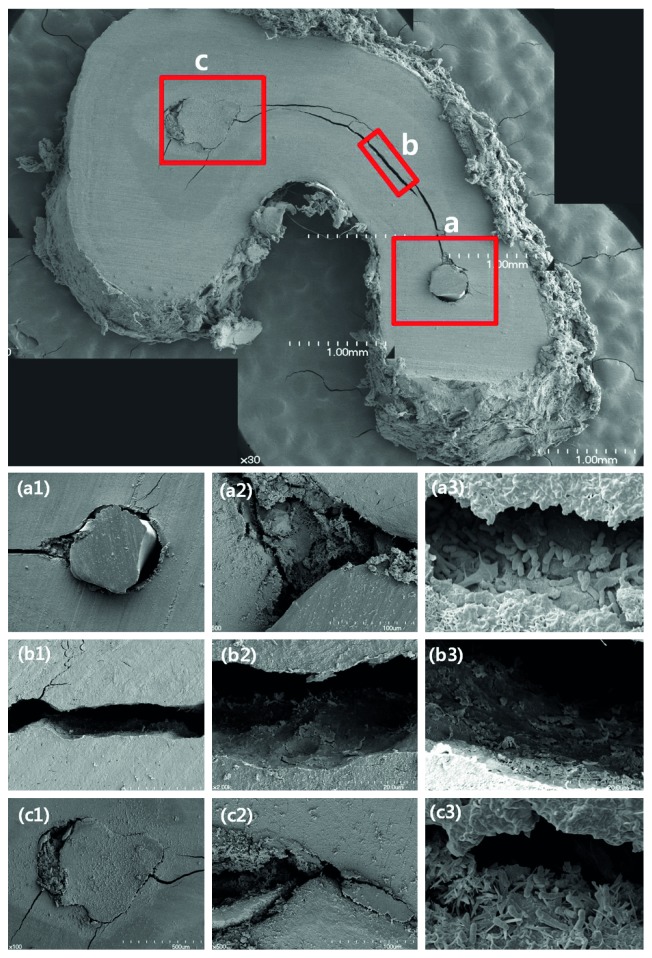
SEM images of resected root tip of C-shaped root canals. (a1) Leakage around the gutta-percha filling area on the distal canal (×100). (a2) Pulp tissue and debris were observed (×500). (a3) Remaining microorganisms were found in the gap between the gutta-percha and the canal wall (×5000). (b1) Isthmus connecting mesiolingual and distal canals (×100). (b2, b3) Pulp tissue and debris were observed. (c1) Leakage around the gutta-percha filling area on the mesiolingual canal (×100). (c2) Pulp tissue and debris were observed (×500). (c3) Remaining microorganisms were found in the gap between the gutta-percha and the canal wall (×5000).

The most common cause of endodontic failure of C-shaped root canals was a leaky canal (45.2%), followed by an isthmus (23.8%), missing canal (9.5%), overfilling (7.1%), and iatrogenic problems (7.1%) (Figure 3, *p* < 0.05).  Figure 4 shows the representative photos of various causes of endodontic failure of C-shaped root canals. SEM images of leaky canal and isthmus of C-shaped root canals are shown in Figure 5.

Mandibular second molars were most frequently involved teeth (90.4%) (Table 1, *p* < 0.05). The most frequently observed root canal configuration was C1 at the coronal level (45.2%) and C3 at the apical 3 mm level (45.2%) (Table 2, *p* < 0.05).

## 4. Discussion

This study analyzed various characteristics and classification of C-shaped root canals and provided the clinical causes of failed previous endodontic treatment of C-shaped root canals by examining the resected root surface with endodontic microscope and SEM.

In this study, mandibular second molars (90.4%) were the most commonly involved teeth followed by maxillary second molars and maxillary first molar with a low prevalence (7.2% and 2.4%, respectively) (Table 1). These results were similar to the results of previous studies [3–6]. The nonsurgical root canal treatment of the mandibular second molar is difficult because of the high prevalence of C-shaped canals, as well as a changing canal configuration according to the root level.

Failure of nonsurgical root canal treatment is due to the presence of residual bacteria or reinfection of a root canal system. Persistent intraradicular infection can be found in missed canals, incomplete instrumentation, ledge or transportation formation, and dentinal tubules or apical deltas [14, 22]. Complex irregularities at the apical level are often reported in C-shaped root canals [1, 3, 4, 6]. In this study, the most frequently observed root canal configuration was C3 (44.7%) at the apical 3 mm level, followed by C1 (31.9%), C2 (17.0%), and C4 (6.4%) (Table 2). These findings were similar to the results of previous studies [3, 4, 23] and clinically important because complete debridement of the C3 configuration with a narrow isthmus using a mechanical preparation is difficult.

The present study showed that the most common cause of endodontic failure of C-shaped root canals was a leaky canal (45.2%) and isthmus (23.8%) (Figure 3). The representative photo of the leaky canal is shown in Figure 4, (c1) and (c2), and the remaining microorganisms were observed in the gap between the gutta-percha and the root canal wall in SEM images (Figure 5, (a3) and (c3)). Microorganisms that have formed biofilms show a higher resistance to antimicrobial treatment. The disruption of biofilms and reduction of microorganisms can be achieved by a combination of mechanical instrumentation, irrigation with various devices, and application of antimicrobial medicaments in the root canal [24–26]. Therefore, agitation using ultrasonic files or a sonic device such as EndoActivator (Dentsply Sirona, York, PA, USA) can help to remove debris and necrotic pulp tissue from a C-shaped root canal system [27].

Ma et al. found that 68% of the canal space remained filled by Ca(OH)_2_ after instrumentation and conventional needle irrigation in the apical third of C-shaped root canals [27]. On the other hand, the proportion filled by Ca(OH)_2_ decreased to 28% and 31% after EndoActivator and ultrasonic irrigation, respectively. However, complete removal of Ca(OH)_2_ in the apical area of C-shaped canals was still difficult, even after using various irrigation devices, according to that study. Other studies have found that a self-adjusting file (SAF) is more effective in C-shaped root canals than other rotary systems [12, 16]. Solomonov et al. found that SAF preparation left 41 ± 14% of the canal wall unaffected by the procedure; however, 66 ± 6% of the canal wall was left unaffected in the ProTaper group [16].

The representative photo of the isthmus is shown in Figure 4, (d1) and (d2), and isthmus connecting the mesiolingual and the distal canals was observed in SEM images (Figure 5, (b1)). Canal filling materials such as gutta-percha and endodontic sealers were not existing, and the untouched pulp tissue and debris were remained in the isthmus (Figure 5, (b2) and (b3)). After chemomechanical disinfection of C-shaped root canals, complete sealing is important for preventing reinfection. Ordinola-Zapata et al. reported that the mean percentage of gutta-percha-filled areas at the apical level was 74.5% because of various anatomical types of C-shaped root canals [28]. In a recent study, the core-carrier technique was the most effective obturation technique in simulated C-shaped canals compared to the lateral condensation and injectable thermoplasticized gutta-percha techniques [29].

To reduce endodontic failure of C-shaped root canals, clinicians should know the accurate morphology of C-shaped root canals, make an effort to remove the pulp tissue and microorganisms, and completely seal the root canal system without voids. The use of a microscope during endodontic procedures and taking CBCT scans provide a better understanding of the various configurations of C-shaped root canals.

## 5. Conclusions

This study demonstrated that C-shaped root canals were most frequently found at the mandibular second molars in a Korean population. The most common causes of endodontic failure of C-shaped root canals were a leaky canal and an isthmus.

## Figures and Tables

**Table 1:** Various factors associated with C-shaped root canals.

	Total, *n* (%)	Fisher's exact test significance
Sex		*p* > 0.05
Male	19 (45.2)	
Female	23 (54.8)	
Age		*p* < 0.05
20–29	11 (26.2)	
30–39	17 (40.5)	
40–49	7 (16.6)	
50–59	6 (14.3)	
60–	1 (2.4)	
Tooth type		*p* < 0.05
Mandibular left second molar	23 (54.7)	
Mandibular right second molar	15 (35.7)	
Maxillary left second molar	1 (2.4)	
Maxillary right second molar	2 (4.8)	
Maxillary right first molar	1 (2.4)	
Periodontal probing depths		*p* < 0.05
<3 mm	25 (59.5)	
4–6 mm	6 (14.3)	
>7 mm	11 (26.2)	
Lesion size (diameter)		*p* < 0.05
<2 mm	15 (35.7)	
2–5 mm	21 (50.0)	
>5 mm	6 (14.3)	

**Table 2:** Classification of C-shaped root canals.

	C1	C2	C3	C4	Total	Fisher's exact test significance
Coronal level (%)	19 (45.2)	15 (35.7)	7 (16.7)	1 (2.4)	42 (100)	*p* < 0.05
Apical 3 mm level (%)	15 (35.7)	7 (16.7)	19 (45.2)	1 (2.4)	42 (100)	*p* < 0.05

## Data Availability

The datasets used and/or analyzed during the current study are available from the corresponding author on reasonable request.
